# Supplementary material for: Evolutionary Diversification of Host-Targeted Bartonella Effectors Proteins Derived from a Conserved FicTA Toxin-Antitoxin Module
Source: Microorganisms. 2021 Jul 31;9(8):1645. doi: 10.3390/microorganisms9081645 (PMC8401265; doi:10.3390/microorganisms9081645)
Supplement: Supplementary file 1 [file microorganisms-09-01645-s001.zip › microorganisms-1306119-supplementary.pdf]

Supplementary Materials

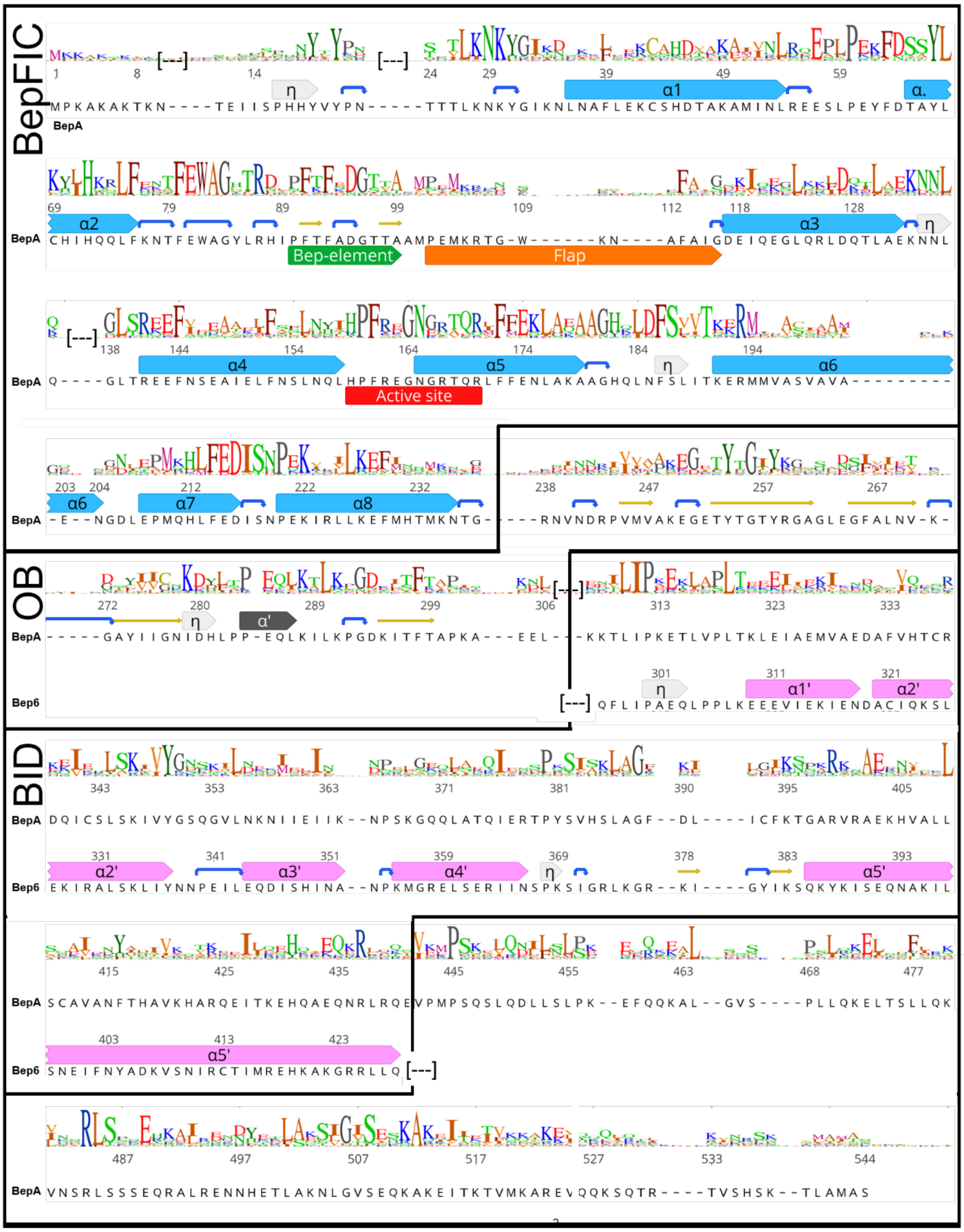

Figure S1. Full-length sequence logo of FIC-BID Beps. Representation as in Fig. 3. Sections corresponding to the BepFIC, OB, and BID domains are indicated at the left. Secondary structure of the FIC and OB domains (helices α1 to α8, α', etc.) has been derived from *Bhe*_BepA (5NH2), secondary structure of the BID domain (helices α1' to α5', etc.) has been derived from *Bro*_Bep6 (4YK1).

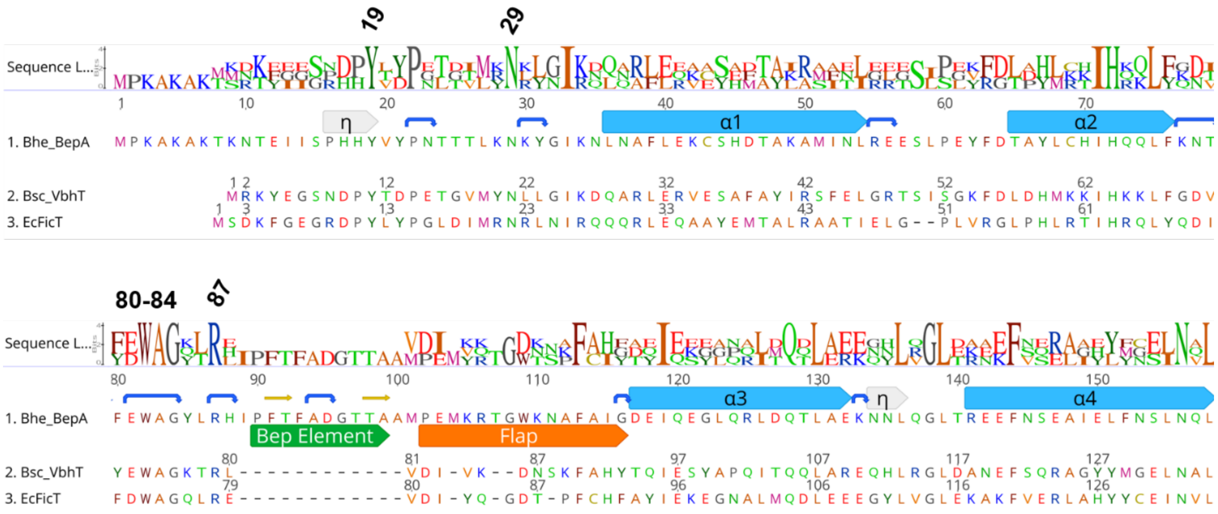

Figure S2. Alignment of *Bhe*_BepA with outgroup members VbhT and *Ec*_FicT. The alignment shows that the N-terminus is conserved, while the Bep element is specific to the Beps.

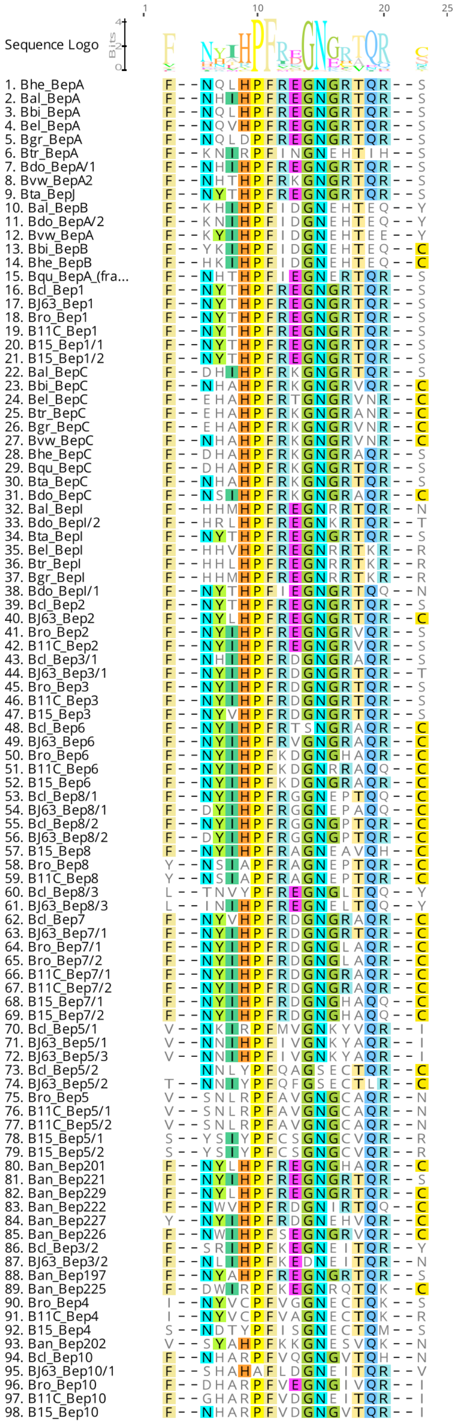

Figure S3. Excerpt of the overall BepFic sequence alignment covering *Bhe*_BepA active site. The non-contiguous segments encompass *Bhe*_BepA residues F113, N156-R170, and S198, see also Fig. 6a. At the right, a green rectangle indicates a canonical FIC motif, a red rectangle an E in position G2 (groups b and c, respectively, in Fig. 6b).

F									
N	0.193								
H	**0.581**	0.202							
G2	**0.446**	0.412	0.284						
E	−0.382	−0.303	−0.233	−0.784					
R1	**0.539**	0.359	**0.543**	**0.557**	−0.589				
Q	0.005	**0.517**	0.060	0.350	−0.212	0.113			
R2	0.089	0.193	0.116	**0.446**	−0.456	**0.407**	0.200		
S	**0.539**	**0.511**	**0.511**	**0.450**	−0.325	**0.544**	0.170	0.181	
	F	N	H	G2	E	R1	Q	R2	S

Figure S4. Correlation (see Methods) between selected residues of the non-contiguous active site motif shown in Fig. 6.

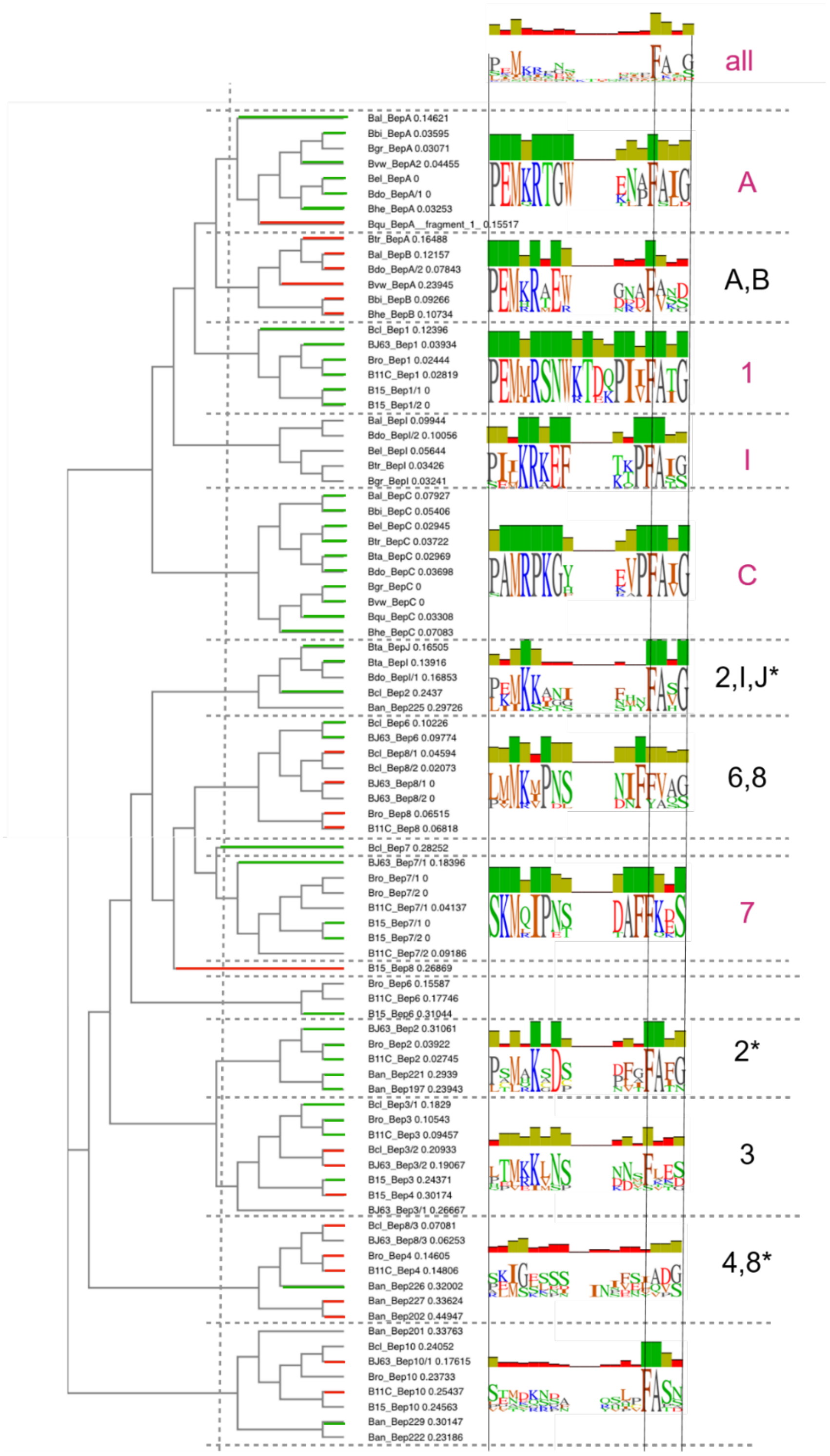

Figure S5. Cladogram derived from all BepFIC flap sequences used in this study (with the exception of the flap-less Bep5 sequences). Branches are labeled according to the sub-clades of their members, asterisks indicate the additional presence of *Ban* members. Horizontal lines colored in green or red indicate Beps with canonical FIC motif or with a glutamate in position G2 (groups b and d, respectively, in Fig. 6b). Names in magenta indicate the major branches as defined in the legend to Fig. 8.

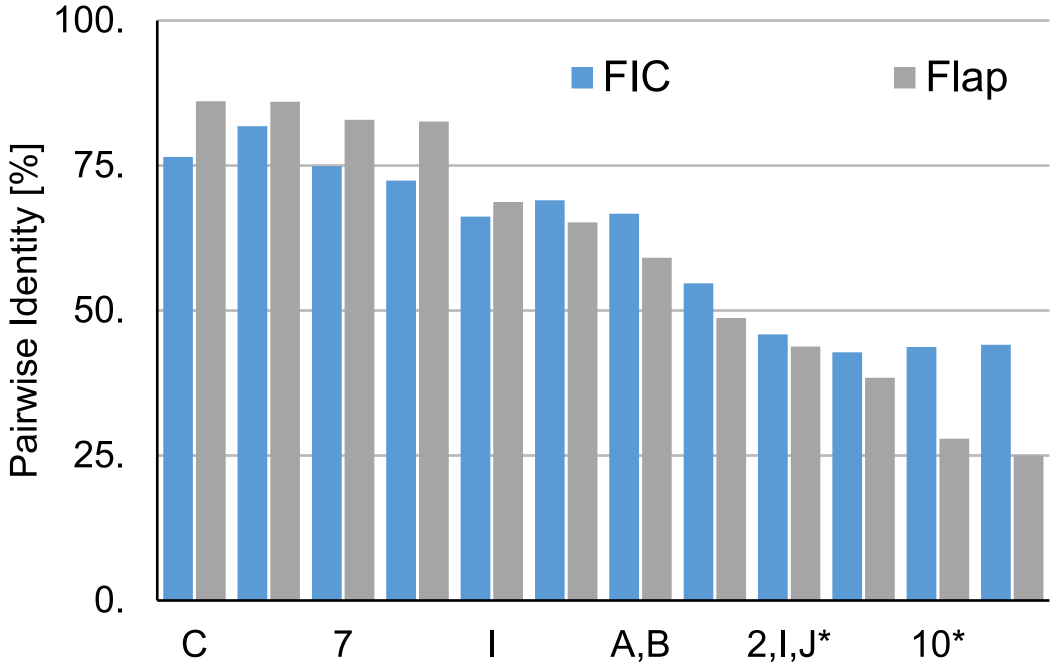

Figure S6. Overall (blue) and flap (green) sequence identity of the flap branches as shown in the cladogram of Fig. S5

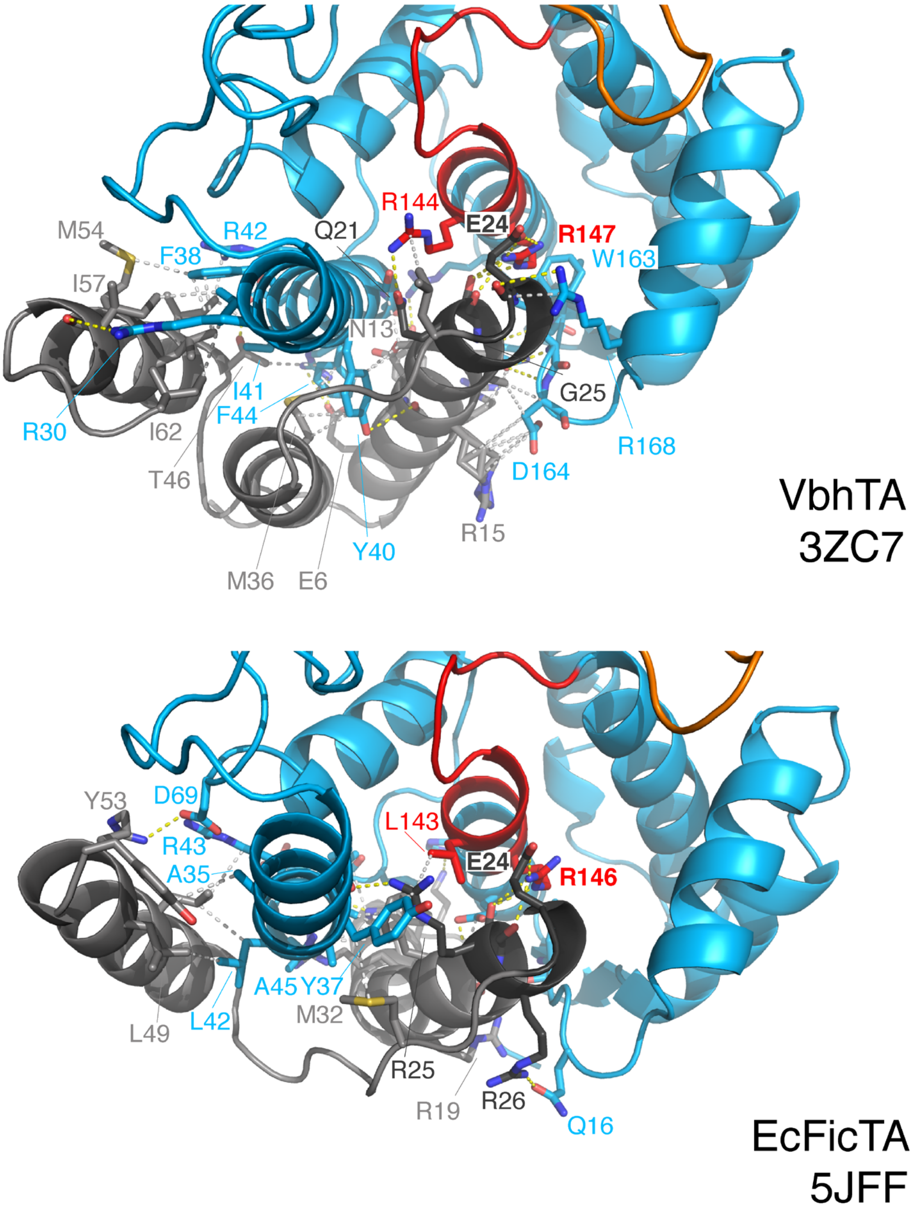

Figure S7. FicTA toxin/antitoxin interaction. The interacting residues between the toxin (blue) and the antitoxin (grey) are shown in light grey indicating apolar interactions and yellow indicating hydrogen bonds. The FIC motif is red and the flap is in orange. (A) VbhTA complex (PBD: 3ZC7). (B) *Ec*_FicAT complex (PBD: 5JFF).

Table S1. List of species abbreviations and genome references.

3-letter code	Organism	NCBI taxonomy ID	Accession number(s)
B11C	*Bartonella* sp. 1-1C	NCBI:txid515256	CP019489
BA13	*Bartonella* sp. A1379B	NCBI:txid1933910	CP019780
B15	*Bartonella* sp. AR 15-3	NCBI:txid545617	MUYE00000000
Bal	*Bartonella alsatica* IBS 382	NCBI:txid1094551	AIME01000001.1 - AIME01000021.1
Ban	*Bartonella ancashensis* 20.00	NCBI:txid1318743	KY583505
BbK	*Bartonella bacilliformis* KC583	NCBI:txid360095	NC_008783
BbV	*Bartonella bacilliformis* Ver075	NCBI:txid1293904	ASIV01000001 - ASIV01000010
Bbi	*Bartonella birtlesii* IBS 325	NCBI:txid1095900	AKIP01000001_1.1 - AKIP01000035_1.35
Bbo	*Bartonella bovis* 91-4	NCBI:txid1094491	AGWA01000001 - AGWA01000019
Bcd	*Bartonella* sp. CDCskunk	NCBI:txid1933905	CP019782
Bcl	*Bartonella clarridgeiae* 73	NCBI:txid696125	NC_014932.1
Bco	*Bartonella* sp. Coyote22sub2	NCBI:txid1933911	CP01978
Bdo	*Bartonella doshiae* NCTC 12862	NCBI:txid1094553	AILV01000001 - AILV01000025
Bel	*Bartonella elizabethae* F9251	NCBI:txid109455	AIMF01000001.1 - AIMF01000049.1
Bgr	*Bartonella grahamii* as4aup	NCBI:txid634504	NC_012846 + NC_012847
Bhe	*Bartonella henselae* Houston-1	NCBI:txid283166	NC_005956
Bhd	*Bartonella* sp. Hoopa Dog 114	n.a.	CP019784
Bhf	*Bartonella* sp. Hoopa Fox 11B	n.a.	CP019783
BJ15	*Bartonella* sp. JB15	NCBI:txid1933906	CP019787
BJ63	*Bartonella* sp. JB63	NCBI:txid1933907	CP019788
Bqu	*Bartonella quintana* Toulouse	NCBI:txid283165	NC_005955
BR60	*Bartonella* sp. Raccoon60	NCBI:txid1933912	CP019786
Bro	*Bartonella rochalimae* ATCC BAA-1498	NCBI:txid685782	FN645455 - FN645467
Bsb	*Bartonella schoenbuchensis* R1	NCBI:txid687861	CP019789 + CP019790
BW12	*Bartonella* sp. SikaDeer WD12.1	n.a.	MUBG00000000
BW16	*Bartonella* sp. SikaDeer WD16.2	n.a.	CP01978
Bta	*Bartonella taylorii* IBS 296	n.a.	MUYW00000000
Btr	*Bartonella tribocorum* CIP 105476	NCBI:txid382640	NC_010160 + NC_010161
Bvw	*Bartonella vinsonii* subsp. *berkhoffii* strain Winnie	NCBI:txid1094497	CP003124

Table S2. List of FIC-BID Beps used in this study.

Name	UNIPROT
**Bal_BepA**	J1IX26
**Bal_BepB**	J0PTT5
**Bal_BepC**	J1IY69
**Bal_BepI**	J1IY65
**Ban_Bep197**	A0A0M3T2K6
**Ban_Bep201**	A0A0M3T2M9
**Ban_Bep202**	A0A0M5KSB7
**Ban_Bep221**	A0A0M3T2N3
**Ban_Bep222**	A0A0M5KSB8
**Ban_Bep225**	A0A0M4LIT1
**Ban_Bep226**	A0A1V0PNG5
**Ban_Bep227**	A0A0M3T2L2
**Ban_Bep229**	A0A0M4LHM1
**Bbi_BepA**	UPI000379BB9C
**Bbi_BepB**	UPI000367D6D4
**Bbi_BepC**	UPI0003824625
**Bcl_Bep1**	E6YFW2
**Bcl_Bep2**	E6YIF3
**Bcl_Bep3/1**	E6YHI2
**Bcl_Bep3/2**	E6YHH2
**Bcl_Bep5/1**	E6YGF5
**Bcl_Bep5/2**	E6YGD8
**Bcl_Bep6**	E6YHH5
**Bcl_Bep7**	E6YHI3
**Bcl_Bep8/1**	E6YHH4
**Bcl_Bep8/2**	E6YHH3
**Bcl_Bep8/3**	E6YHH8
**Bcl_Bep10**	E6YIM4
**Bdo_BepA/1**	J0YV99
**Bdo_BepA/2**	J0YVD3
**Bdo_BepC**	J1J5M3
**Bdo_BepI/1**	J1J4K1
**Bdo_BepI/2**	J0Q230
**Bel_BepA**	J0R905
**Bel_BepC**	J0R175
**Bel_BepI**	J0ZTN1
**Bgr_BepA**	C6AES4
**Bgr_BepC**	C6AES7
**Bgr_BepI**	C6AET2
**Bhe_BepA**	Q5QT06
**Bhe_BepB**	Q5QT04
**Bhe_BepC**	Q5QT03
**Bqu_BepA (fragment 1)**	no accession in this strain
**Bqu_BepA (fragment 2)**	no accession in this strain
**Bqu_BepC**	A0A0H3LV04
**Bro_Bep1**	E6YJU0
**Bro_Bep2**	E6YMI0
**Bro_Bep3**	E6YLF6
**Bro_Bep4**	E6YLB1
**Bro_Bep5**	E6YKB8

Name	UNIPROT
**Bro_Bep6**	E6YLF3
**Bro_Bep7/1**	E6YLF1
**Bro_Bep7/2**	E6YLF7
**Bro_Bep8**	E6YLF2
**Bro_Bep10**	E6YMR3
**B11C_Bep1**	E6YTB2
**B11C_Bep2**	E6YW78
**B11C_Bep3**	E6YV82
**B11C_Bep4**	E6YV21
**B11C_Bep5/1**	E6YVB0
**B11C_Bep5/2**	E6YU24
**B11C_Bep6**	E6YV78
**B11C_Bep7/1**	E6YV76
**B11C_Bep7/2**	E6YV83
**B11C_Bep8**	E6YV77
**B11C_Bep10**	E6YWF1
**B15_Bep1/1**	E6YNY8
**B15_Bep1/2**	E6YNY8
**B15_Bep3**	E6YQP7
**B15_Bep4**	E6YQI1
**B15_Bep5/1**	E6YQT0
**B15_Bep5/2**	E6YPK5
**B15_Bep6**	E6YQQ0
**B15_Bep7/1**	A0A1T3BS91
**B15_Bep7/2**	E6YQQ2
**B15_Bep8**	E6YQQ1
**B15_Bep10**	E6YS53
**BJ63_Bep1**	UPI0009995D5F
**BJ63_Bep2**	UPI00099A7C8C
**BJ63_Bep3/1**	UPI00099B008B
**BJ63_Bep3/2**	UPI00099A7C8A
**BJ63_Bep5/1**	UPI0009C1F7B7
**BJ63_Bep5/2**	UPI00099A9131
**BJ63_Bep5/3**	UPI0009C348C6
**BJ63_Bep6**	UPI00099B1F53
**BJ63_Bep7/1**	UPI0009999BB7
**BJ63_Bep8/1**	UPI00099A6FF8
**BJ63_Bep8/2**	UPI000999E53B
**BJ63_Bep8/3**	UPI00099AB2FD
**BJ63_Bep10/1**	UPI000999CC63
**Bta_BepC**	UPI00026E5F09
**Bta_BepI**	UPI00026E5F06
**Bta_BepJ**	UPI00026E5F0C
**Btr_BepA**	A9IWP4
**Btr_BepC**	A9IWP7
**Btr_BepI**	A9IWQ5
**Bvw_BepA**	N6UQE3
**Bvw_BepA2**	N6VKZ4
**Bvw_BepC**	N6UWX1

Table S3. Data collection and refinement statistics.

	*Bhe*_BepA /BiaA$_{L4}$	*Bqu*_BepC/ ADP	*Bqu*_BepC	*Btr*_BepC/ AMPPNP	*Bcl*_Bep1	*Bcl*_Bep5	*Bsp15*_Bep8	*B11C*_Bep8
**Data collection**								
Space group	$P\ 2_1\ 2_1\ 2$	$P\ 2_1\ 2_1\ 2_1$	$P\ 2_1$	$P\ 2_1\ 2_1\ 2$	$P\ 2_1\ 2_1\ 2$	$P\ 2_1\ 2_1\ 2$	$P\ 2_1$	$P\ 2_1$
a, b, c (Å)	48.03, 56.15, 136.60	40.45, 64.07, 97.60	57.56, 43.63, 88.76	59.33, 92.02, 45.84	73.19, 97.85, 49.11	99.27, 122.86, 143.71	40.24, 47.48, 67.97	55.81, 324.16, 86.13
β (⁰)	90.00	90.00	91.53	90.00	90.00	90.00	105.64	109.24
Resolution (Å)*	2.32 (2.40-2.32)	1.55 (1.59-1.59)	2.00 (2.05-2.00)	1.70 (1.74-1.70)	1.90 (1.95-1.90)	2.95 (3.03-2.95)	1.85 (1.90-1.85)	2.35 (2.41-2.35)
Unique reflections	121245	37518	30109	28113	28414	37608	21213	118369
Completeness (%)	94 (84)	99.6 (99.3)	99.7 (99.9)	99.2 (92.1)	99.6 (99.7)	99.7 (100.0)	99.6 (99.8)	99.0 (99.2)
I/σ(I)	18.1 (2.6)	22.7 (3.5)	8.2 (3.1)	20.9 (2.1)	27.8 (3.7)	17.4 (3.7)	18.9 (2.3)	13.3 (2.4)
Redundancy	7.7 (6.6)	6.1 (6.0)	3.7 (3.4)	4.5 (2.1)	5.2 (5.3)	6.1 (6.3)	4.1 (3.8)	3.7 (3.9)
R^a_{sym} (%)	9.5 (66.0)	4.6 (49.0)	11.4 (38.6)	5.0 (46.5)	3.6 (52.2)	7.5 (51.6)	5.9 (55.2)	8.0 (56.9)
**Refinement**								
R^b_{work}/R^c_{free} (%)	26.2 (32.8)	17.2 (19.4)	18.3 (23.5)	16.4 (19.3)	17.2 (21.2)	23.5 (27.9)	16.4 (21.1)	24.2 (25.9)
Rmsd from ideal values								
Bond length (Å)	0.013	0.010	0.017	0.006	0.013	0.002	0.014	0.009
Bond angles (°)	1.2	1.5	1.8	1.0	1.5	0.54	1.5	1.2
No. of atoms								
Protein	2756	1770	3516	1661	2185	8683	1839	16440
Ligands	1	28	14	45	8	12	4	8
Metals	1	1	0	0	0	0	0	0
Water	83	257	325	231	161	8	271	267
Average B-factor (Å²)								
Protein	48.2	19.9	18.8	19.6	34.5	82.1	23.1	50.8
Ligands	62.2	28.0	31.8	32.2	58.4	89.7	32.3	60.2
Metals	62.2	27.15	NA	NA	NA	NA	NA	NA
Water	45.4	41.3	26.3	32.6	43.4	59.8	31.9	36.7
Ramachandran statistics (%)								
Favored regions	98	100	99.5	100	100	98	99	98
Allowed regions	1.8	0	0.5	0	0	2	1	2
Disallowed regions	0.29	0	.	0	0	0	0	1
**PDB ID**	5NH2	4N67	4LU4	4WGJ	4NPS	4XI8	4M16	4PY3

* The values recorded in parentheses are those for the highest resolution shell

[a] R_{sym} = Σ |I - <I>| / Σ |I|, where I is the observed intensity and <I> is the average intensity of several symmetry-related observations.

[b] R_{work} = Σ ||Fo| - |Fc|| / Σ |Fo|, where Fo and Fc are the observed and calculated structure factors, respectively.

[c] R_{free} = Σ ||Fo| - |Fc|| / Σ |Fo| for 7% of the data not used at any stage of the structural refinement.
